# The Expression Profile of Phosphatidylinositol in High Spatial Resolution Imaging Mass Spectrometry as a Potential Biomarker for Prostate Cancer

**DOI:** 10.1371/journal.pone.0090242

**Published:** 2014-02-28

**Authors:** Takayuki Goto, Naoki Terada, Takahiro Inoue, Kenji Nakayama, Yoshiyuki Okada, Takeshi Yoshikawa, Yu Miyazaki, Masayuki Uegaki, Shinji Sumiyoshi, Takashi Kobayashi, Tomomi Kamba, Koji Yoshimura, Osamu Ogawa

**Affiliations:** 1 Department of Urology, Graduate School of Medicine, Kyoto University, Kyoto, Japan; 2 Department of Diagnostic Pathology, Kyoto University Hospital, Kyoto, Japan; Yokohama City University School of Medicine, Japan

## Abstract

High-resolution matrix-assisted laser desorption/ionization imaging mass spectrometry (HR-MALDI-IMS) is an emerging application for the comprehensive and detailed analysis of the spatial distribution of ionized molecules in situ on tissue slides. HR-MALDI-IMS in negative mode in a mass range of m/z 500–1000 was performed on optimal cutting temperature (OCT) compound-embedded human prostate tissue samples obtained from patients with prostate cancer at the time of radical prostatectomy. HR-MALDI-IMS analysis of the 14 samples in the discovery set identified 26 molecules as highly expressed in the prostate. Tandem mass spectrometry (MS/MS) showed that these molecules included 14 phosphatidylinositols (PIs), 3 phosphatidylethanolamines (PEs) and 3 phosphatidic acids (PAs). Among the PIs, the expression of PI(18:0/18:1), PI(18:0/20:3) and PI(18:0/20:2) were significantly higher in cancer tissue than in benign epithelium. A biomarker algorithm for prostate cancer was formulated by analyzing the expression profiles of PIs in cancer tissue and benign epithelium of the discovery set using orthogonal partial least squares discriminant analysis (OPLS-DA). The sensitivity and specificity of this algorithm for prostate cancer diagnosis in the 24 validation set samples were 87.5 and 91.7%, respectively. In conclusion, HR-MALDI-IMS identified several PIs as being more highly expressed in prostate cancer than benign prostate epithelium. These differences in PI expression profiles may serve as a novel diagnostic tool for prostate cancer.

## Introduction

Prostate cancer is one of the most common cancers and the major leading cause of cancer-related deaths in men [1]. Because of its clinical and histological heterogeneity, the mechanisms underlying prostate cancer development have not yet been determined. Lipid metabolism may play an important role in human carcinogenesis by affecting numerous cellular processes, including cell growth, proliferation, differentiation and motility [2]–[4]. The expression patterns of several phospholipids have been reported to differ in prostate cancer and benign prostate tissue [5]. Phosphatidylinositols (PIs), a major class of phospholipids, are involved in intracellular signal transduction [6], [7]. In particular, the PI3-kinase pathway, which regulates many cellular functions, including lipid metabolism, is frequently mutated or activated in prostate cancer [8], [9]. Unlike other phospholipids, PI profiles show specific patterns in mammalian cells, being affected by several acyltransferases in the remodeling pathway (Lands' pathway) [10], [11]. The PI profiles of human breast cancer tissues have been shown to differ from those of normal mammary glands [12]. To date, however, PI profiles have not been analyzed in prostate cancer tissues.

Matrix assisted laser desorption/ionization imaging mass spectrometry (MALDI-IMS) is a new modality that facilitates the acquisition of comprehensive mass spectra directly from tissue specimens and provides reconstructed density maps of detected ions [13]. Prostate cancer, however, is multifocal and surrounded by benign prostate epithelium or stroma, making it difficult to identify cancer specific lesions by conventional MALDI-IMS. The spatial resolution of this technique was recently improved, to less than 10 µm, facilitating a detailed two-dimensional analysis of phospholipids [12], [14]–[18]. As most prostate cancer forms glands and the diameter of a single gland is no smaller than 50 µm, the 10 µm pitch of high-resolution matrix-assisted laser desorption/ionization imaging mass spectrometry (HR-MALDI-IMS) is sufficient to clearly visualize prostate cancer lesions.

Frozen tissue samples embedded in optimal cutting temperature (OCT) compound are preserved in many clinical laboratories and are frequently used in cancer research. As a result of contamination problem, however, fresh frozen samples without OCT compound have been used in IMS lipid research [19], [20]. The use of samples stored without OCT compound is limited, because of the formation of ice crystals and difficulty making tissue sections.

In this study, we established an in situ system using HR-MALDI-IMS to analyze phospholipid expression patterns of prostate tissue samples embedded in OCT compound. This method identified several phospholipids as being more highly expressed in prostate cancer than in adjacent benign epithelium. We therefore focused on the expression profile of PIs and evaluated their potential to distinguish prostate cancer from benign epithelium. To our knowledge, this study is the first to use HR-MALDI-IMS to investigate phospholipid expression patterns in prostate tissue samples embedded in OCT compound, and to successfully identify differences in PI expression profiles in prostate cancer.

## Materials and Methods

### Ethics statement

All experiments involving laboratory animals were performed in accordance with the Guidelines for Animal Experiments of Kyoto University. The protocol was approved by the Committee on the Ethics of Animal Experiments of Kyoto University (Permit Number: 13331).

Clinical materials were used after written informed consent was obtained, according to protocols approved by the institutional review board of Kyoto University Hospital.

### Preparation of OCT compound embedded tissue samples

We previously established a novel mouse xenograft model of human prostate cancer, called KUCaP-2 [21]. These xenograft tissues were used to determine the conditions appropriate for the HR-MALDI-IMS system to analyze human prostate tissue samples embedded in OCT compound. Xenograft tumors were each divided into two pieces, with one embedded whole in OCT compound (Tissue-Tek®; Sakura Finetek, Torrance, CA, USA), without sucrose treatment to avoid the influence of fixation, and the other immediately frozen in liquid nitrogen to avoid degradation of biomolecules. Both samples were stored at −80°C.

The patient cohort consisted of 38 Japanese patients with clinically localized prostate cancer who underwent radical prostatectomy at Kyoto University Hospital from 2005 to 2008. Prostate tissue slices 5 mm thick were harvested immediately after removal and embedded in OCT compound, without sucrose treatment and frozen at −80°C. All frozen blocks yielded sections containing cancer tissue and benign epithelium.

### Histological evaluation and matrix coating of prostate tissue samples

After maximum removal of OCT compound, the tissue samples were cryosectioned on a cryostat (CM1850; Leica, Wetzler, Germany) at −20°C. Cryosections 5 µm thick were mounted onto glass slides (MAS coat; Matsunami, Osaka, Japan) for hematoxylin and eosin (H&E) staining. All slides were evaluated by a single pathologist (S.S.) to determine tissue morphology and as a guide for HR-MALDI-IMS analysis. Additional serial sections at 10 µm were mounted onto indium-tin oxide–coated (ITO) glass slides (Sigma-Aldrich, St Louis, MO, USA) and used for HR-MALDI-IMS analysis. OCT compound-embedded and fresh frozen xenograft samples were mounted onto the same slide, whereas each clinical prostate tissue sample of approximately 7.5×7.5 mm was mounted onto a single slide. Each section was coated with 9-aminoacridine hemihydrates (9-AA) (Acros Organics, Geel, Belgium), which served as the matrix for MALDI-MS. Each slide was anchored in vacuum deposition equipment (SVC-700TM/700-2; Sanyu Electron, Tokyo, Japan) and coated with a 9-AA matrix layer obtained by sublimation at 220°C. The time required for the vapor deposition process was 8 min; the thickness of the 9-AA layer deposited on the slide was not assessed. The sections assessed by HR-MALDI-IMS were also stained with H&E and assessed by the pathologist. For H&E staining after HR-MALDI-IMS analysis, 9-AA was removed from the slides by dipping them in methanol for 30 s.

### HR-MALDI-IMS and MS/MS analysis

HR-MALDI-IMS analysis was performed on a high-resolution microscopic imaging mass spectrometer (RK27-4050C; Shimadzu, Kyoto, Japan; the prototype model of iMScope) equipped with a 355-nm Nd:YAG laser. Mass spectrometry data were acquired in negative mode in the mass range of m/z 500–1000 using an external calibration method with mass resolving power 10,000 at m/z 1000. A region of interest (ROI), approximately 2000×2000 µm in size and containing cancer tissue, benign epithelium and stroma, was randomly determined from the microscopic view of each slide and mass spectra were obtained at a spatial resolution of 10 µm. The ROI was reconfirmed by the 10 µm thick sample stained with H&E after HR-MADLI-IMS measurement. The same instrument was used for tandem mass spectrometry (MS/MS) analysis; the lipid class and fatty acid composition of the observed peaks were based on the spectral patterns of the ion peaks of the products. The Human Metabolome Database (http://www.hmdb.ca/) and Nature Lipidomics Gateway (http://www.lipidmaps.org/) were used for referencing. Of the 38 samples, 14 were randomly selected as a discovery set and used to identify molecules highly expressed in prostate tissues and differentially expressed in prostate cancer and benign epithelium. The other 24 samples were used as a validation set and the expression profiles of the identified molecules were statistically analyzed and their utility as molecular markers for prostate cancer diagnosis was evaluated.

### Data processing and statistical analysis of HR-MALDI-IMS results

Using SIMtools software (in-house software; Shimadzu Corporation, Kyoto, Japan), the mass profiles were normalized relative to the total ion current to eliminate variations in ionization efficiency, and used for all imaging and statistical analyses. Ion images were visualized using Biomap software (Novartis, Basel, Switzerland). Mann-Whitney (M-W) U tests were used to compare factors between fresh frozen tissue and OCT embedded tissue or between cancer and benign epithelium. To evaluate PI expression profiles, the normalized data were imported into SIMCA 13.0.3 software (Umetrics AB, Umeå, Sweden). Principal component analysis (PCA) was used for unsupervised multivariate analyses and orthogonal partial least squares discriminant analysis (OPLS-DA) was used for supervised multivariate analyses. PCA and OPLS-DA models are depicted as score plots (principal component [PC1, PC2]), which display any intrinsic grouping of samples based on spectral variation. In OPLS-DA analysis, potential biomarkers were selected based on the S-plot. The plot of the covariance versus the correlation in conjunction with the variable trend plots resulted in easier visualization of the data. The variables that changed most were plotted at the top or bottom of the “S” shaped plot, and those that did not vary significantly were plotted in the middle. The algorithm to differentiate cancer from benign epithelium was also established by OPLS-DA, with an optimal cutoff point defined as 0.5. The predictive power of the algorithm was also tested using the area under the receiver operator characteristic (ROC) curve. Sensitivity was defined as the proportion of actual cancer regions correctly identified as such, and specificity was defined as the proportion of actual benign epithelium regions correctly identified as such. All statistical analyses were performed using SIMCA 13.0.3 software or SPSS version 11.0, with p<0.05 considered statistically significant.

## Results

### Prostate cancer tissues embedded in OCT compound are suitable for HR-MALDI-IMS

**Figure 1:**
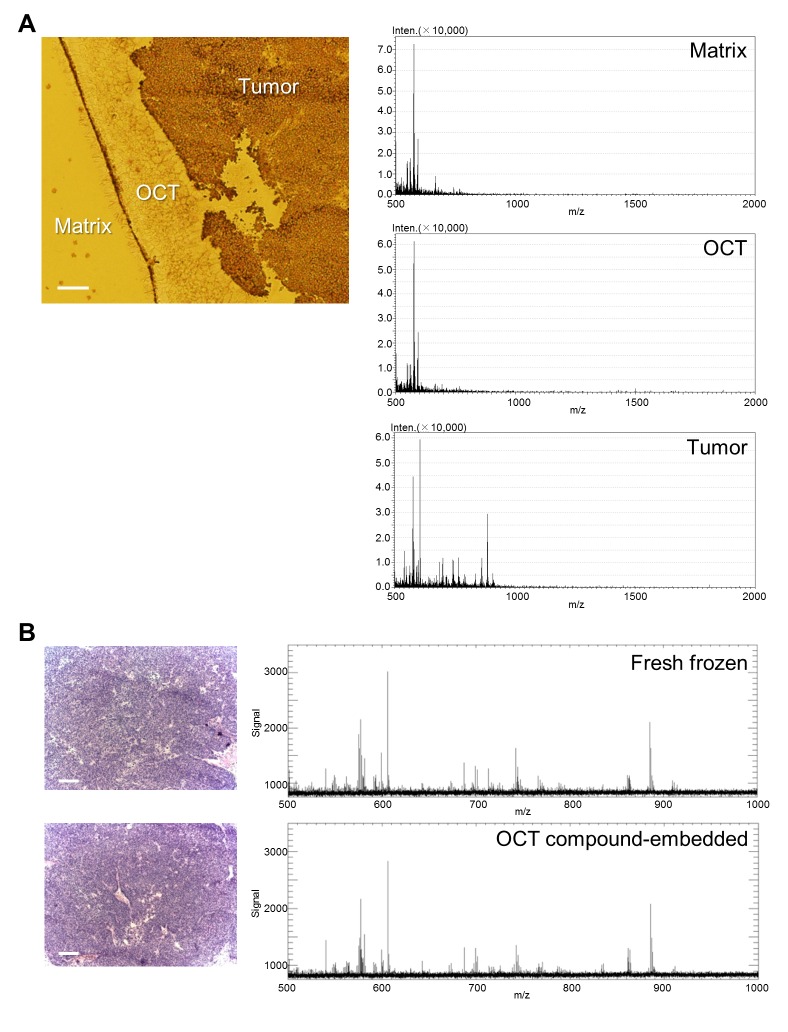
Direct tissue mass spectrometric analysis of prostate cancer xenograft tissues. Matrix coated tissue was subjected to negative ion mode HR-MALDI-IMS. A, Resulting averaged mass spectra of each region (upper panel, matrix; middle panel, OCT+matrix; lower panel, tumor + matrix) in the mass range of m/z 500–2000. The x axes show m/z, and the y axes show the signal intensity of mass spectra. The corresponding optical images are also shown. The scale bar represents 200 µm. B, H&E images of fresh frozen (upper panel) and OCT compound embedded (lower panel) xenograft tumor specimens. The scale bar represents 200 µm. Resulting averaged mass spectra of each xenograft tumor tissue (upper, fresh frozen; lower, OCT compound embedded) in the mass range of m/z 500–1000.

To evaluate the suitability of prostate cancer tissue samples embedded in OCT compound for HR-MALDI-IMS, we used a mouse xenograft model of human prostate cancer (KUCaP-2). The mass spectra on OCT without tumor tissues showed the same peaks as the mass spectra on the 9-AA matrix alone, indicating that OCT did not interfere with the HR-MALDI-IMS analysis in negative mode (Figure 1A). Moreover, in the mass range of m/z 500–1000, there were almost no significant differences in the spectral patterns of OCT compound embedded samples and fresh frozen samples (Figure 1B, Table S1). These results indicated that prostate cancer tissues embedded in OCT compound are suitable for HR-MALDI-IMS analyses in negative mode.

### Twenty phospholipids were identified as highly expressed in human prostate tissues

**Figure 2:**
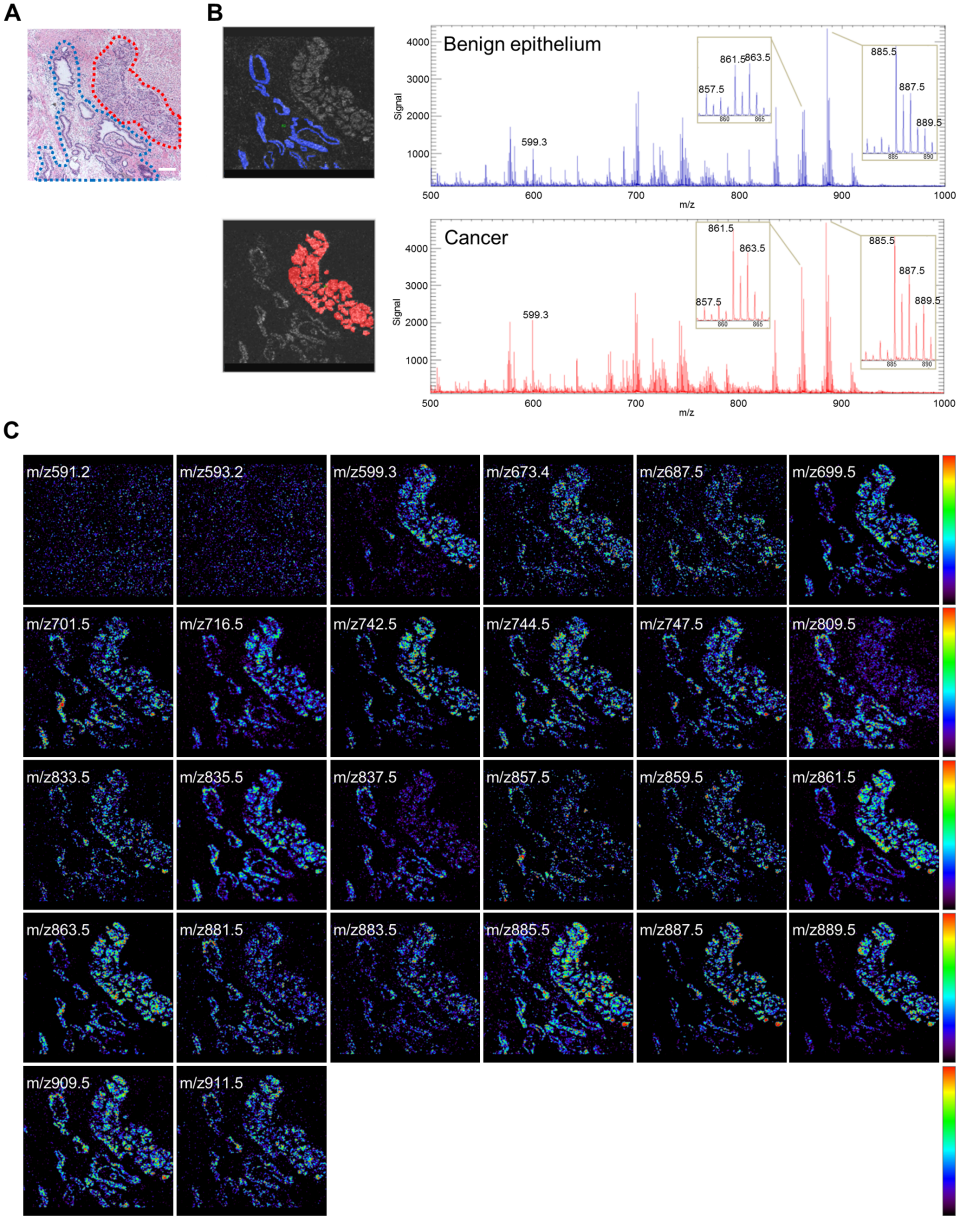
Direct tissue mass spectrometric analysis of human prostate tissue (Case 1). Matrix coated tissue was assessed by negative ion mode HR-MALDI-IMS in the mass range of m/z 500–1000. A, H&E stained human prostate tissue specimen containing defined areas of benign epithelium (blue) and prostate cancer (red). The scale bar represents 200 µm. B, Regions of interest and resulting averaged masses of benign epithelium (upper panel) and prostate cancer (lower panel). The x- and y-axes shows m/z and the signal intensity normalized to total ion current, respectively. C, Mass spectrometry image showing the distribution of 26 common m/z species.

Human prostate tissue samples embedded in OCT compound were analyzed by HR-MALDI-IMS in negative mode in the mass range of m/z 500–1000 (Figure 2A, B). The characteristics of the 38 included patients are shown in Table 1. Of the 38 samples, 14 were used in the discovery set and 24 in the validation set. ROIs that included both cancer and benign epithelium were randomly selected in the 14 discovery set samples. The top 100 peaks of the mass spectra were analyzed in each sample. Excluding matrix and isotopic peaks, 26 peaks were consistently detected in 12 or more of the 14 samples (Table S2). The ion images were clearly visualized on prostate cancer and benign epithelium using HR-MALDI-IMS focusing on these 26 m/z species (Figure 2C). MS/MS analysis could identify the structure of these molecules by analyzing the peaks of their precursor ions (Figure S1). Of the 26 peaks, 20 were identified as phospholipids (Table S3), with 14 being lysophosphatidylinositol (LPI) and PIs (m/z 599.3, 809.5, 833.5, 835.5, 837.5, 857.5, 859.5, 861.5, 863.5, 883.5, 885.5, 887.5, 889.5, and 909.5), three being phosphatidylethanolamines (PEs: m/z 716.5, 742.5, and 744.5) and three being phosphatidic acids (PAs: m/z 673.4, 699.5, and 701.5). The observed saturated fatty acids contained in these phospholipids were C16:0 and C18:0, and the unsaturated fatty acids were C18:1, C18:2, C20:2, C20:3, C20:4, and C22:6.

**Table 1:** Clinical and pathological characteristics of the patients resected for prostate cancer.

Category	Subcategory	Total	Discovery set	Validation set
Number of patients		38	14	24
Mean ± SD age, yr		66.0±7.2	63.3±8.1	67.5±6.5
Mean ± SD preoperative PSA, ng/mL		8.62±4.38	8.24±4.82	8.84±4.30
Mean ± SD prostate weight, g		39.1±13.0	42.8±14.6	37.1±12.2
Gleason scores, n (%)	<7	16(42)	7(50)	9(38)
	7	14(37)	4(29)	10(42)
	>7	8(21)	3(21)	5(21)
Pathological stage, n (%)	pT2	26(68)	12(86)	14(58)
	pT3	12(32)	2(14)	10(42)
Surgical margins, n (%)	Negative	20(53)	8(57)	12(50)
	Positive	18(47)	6(43)	12(50)

Abbreviations: SD, standard deviation. PSA, prostate specific antigen.

### PI expression profiles differ in prostate cancer and benign epithelium

**Figure 3:**
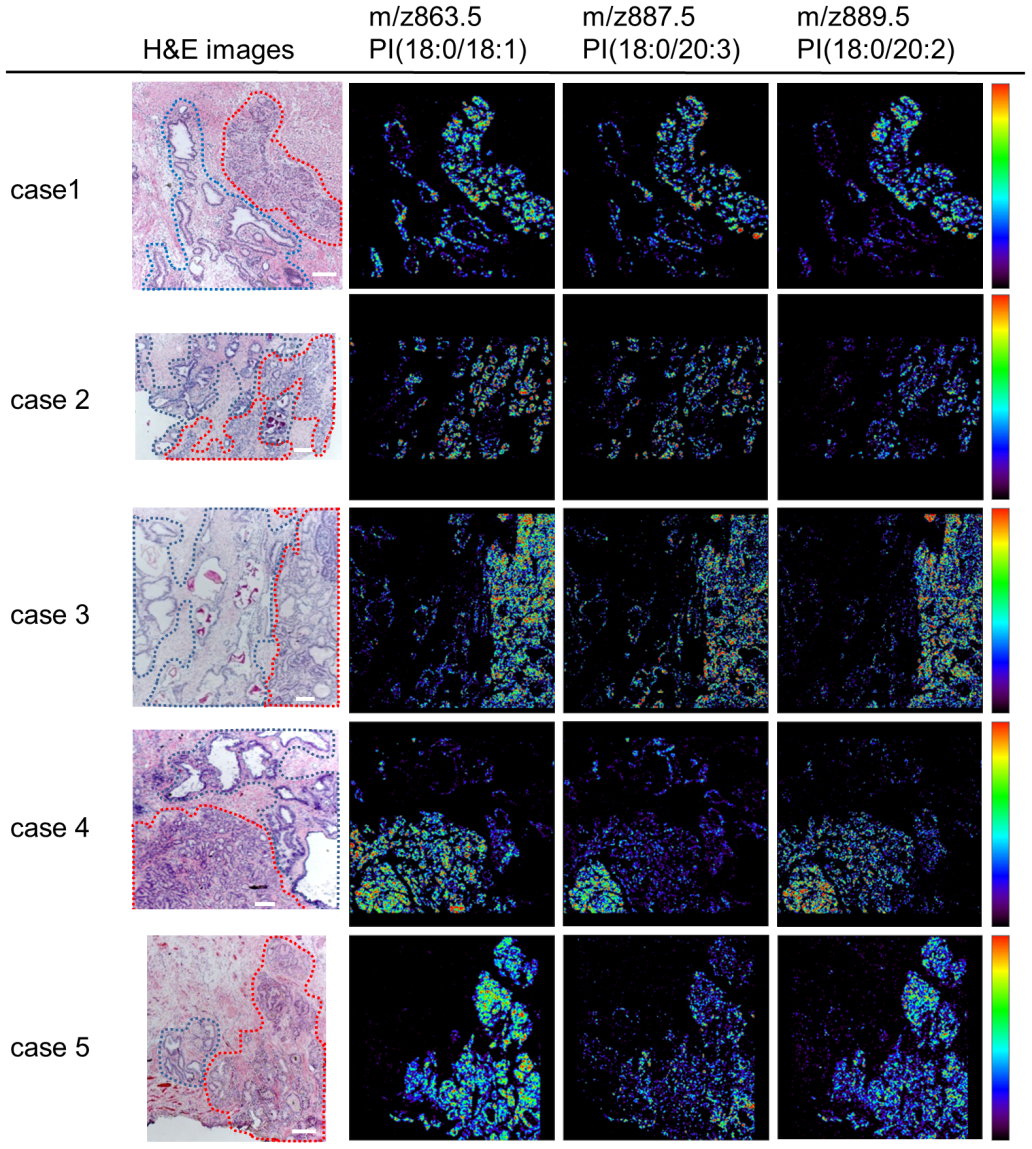
Visualization of molecular distribution of 3 PIs highly expressed in cancer tissue. H&E stained and mass spectrometry images of samples from 5 cases in the discovery set. H&E stained images show defined areas of benign epithelium (blue) and prostate cancer (red). The scale bar represents 200 µm. Mass spectrometry images show the representative distribution of m/z 863.5(PI[18:0/18:1]), 887.5(PI[18:0/20:3]) and 889.5(PI[18:0/20:2]), which were more highly expressed in cancer than in benign epithelium in the discovery set.

The phospholipids most frequently detected by HR-MALDI-IMS analyses in prostate tissue samples were PIs. Therefore we focused on the PI expression profiles in prostate cancer and benign epithelium. A comparison of the signal intensity of these 14 PIs in cancer and benign epithelium in the discovery set samples showed that the expression of PI(18:0/18:1), PI(18:0/20:3) and PI(18:0/20:2) was significantly higher in cancer than in benign epithelium (Table 2). Representative visualizations of the distribution of these PIs in the HR-MALDI-IMS analyses indicated that their levels of expression were similarly higher in cancer than in benign epithelium (Figure 3).

**Table 2:** Averaged signal intensity of all detected PIs in the discovery set.

	m/z	Detected Lipid Species	Benign epithelium, n = 14		Cancer, n = 14		p^*^
			Mean	SD	Mean	SD	Value
A	m/z599.3	PI(18:0/OH)	1410.0	505.1	2032.1	885.0	0.1035
B	m/z809.5	PI(16:0/16:0)	1122.9	413.1	1147.6	648.4	0.4013
C	m/z833.5	PI(16:0/18:2)	864.2	302.6	804.7	277.7	0.8743
D	m/z835.5	PI(16:0/18:1)	3144.0	1255.9	3431.1	1004.5	0.4274
E	m/z837.5	PI(16:0/18:0)	1094.4	405.6	1391.2	530.6	0.0939
F	m/z857.5	PI(16:0/20:4)	1097.6	429.5	830.0	318.5	0.0939
G	m/z859.5	PI(16:0/20:3)	1078.5	374.8	948.9	348.6	0.3761
H	m/z861.5	PI(18:0/18:2)	1887.4	738.3	2229.7	898.4	0.3519
I	m/z863.5	PI(18:0/18:1)	2178.2	831.4	3439.1	1084.0	0.0049
J	m/z883.5	PI(18:1/20:4)	978.0	314.6	861.5	321.3	0.4013
K	m/z885.5	PI(18:0/20:4)	4008.9	1463.0	4002.9	2024.2	0.8036
L	m/z887.5	PI(18:0/20:3)	2364.2	856.8	3563.6	1711.8	0.0212
M	m/z889.5	PI(18:0/20:2)	1133.7	407.2	1708.1	522.5	0.0079
N	m/z909.5	PI(18:0/22:6)	1166.0	472.9	1530.6	567.8	0.0939

Normalized to total ion current and excluding matrix and isotopic peaks. Abbreviations: SD, standard deviation. *Mann Whitney-U test.

**Figure 4:**
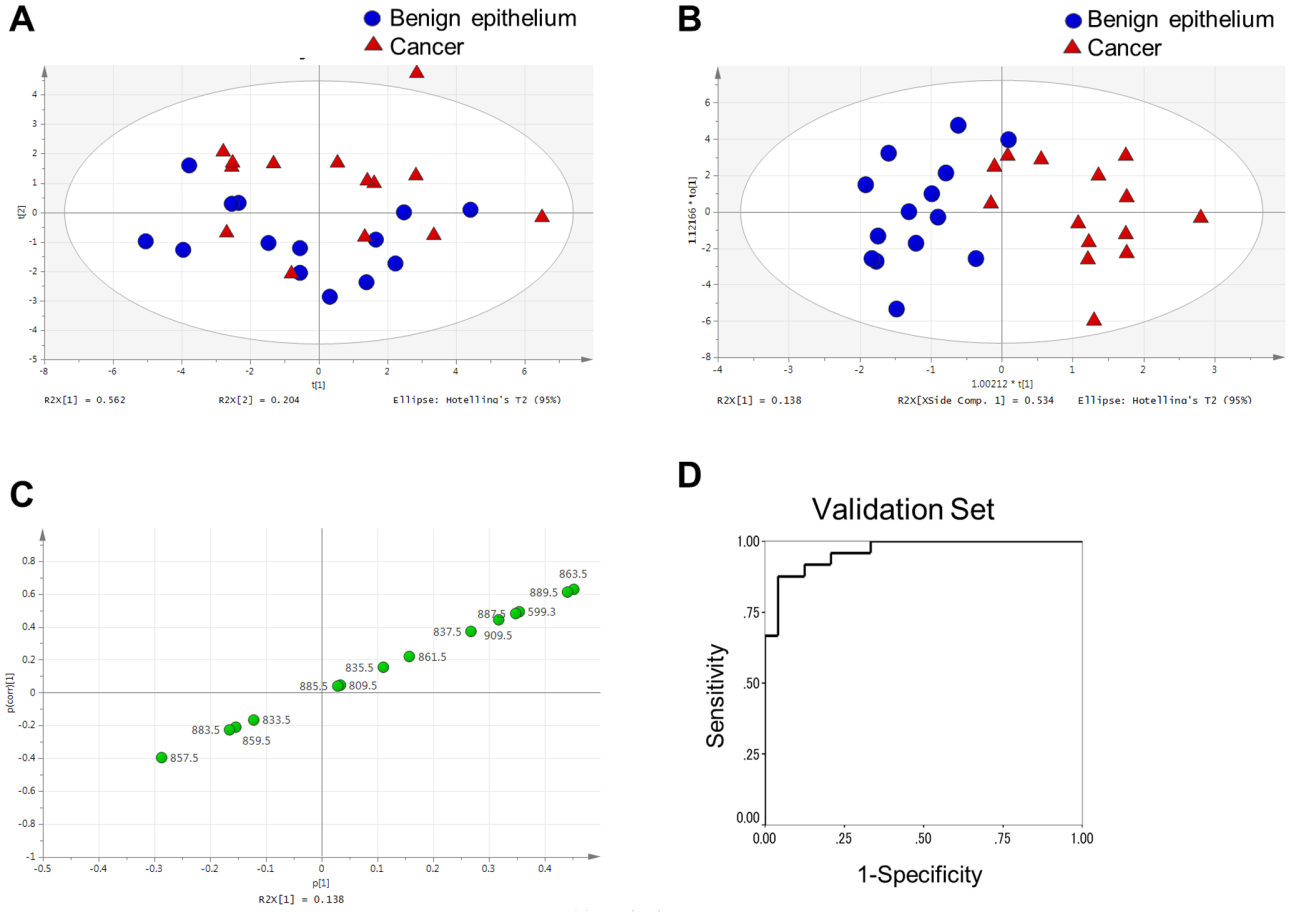
Multivariate analysis of the PI expression profile of prostate cancer and benign epithelium. A, PCA score plot showing the discrimination between cancer and benign epithelium in the discovery set. B, OPLS-DA score plot showing a clear discrimination between cancer and benign epithelium in the discovery set. C, OPLS-DA S-plot corresponding to the score plot shown in (B). The number next to each PI shows its m/z value. D, ROC curve showing the ability of the biomarker algorithm to distinguish prostate cancer from benign epithelium in the validation set.

To assess global differences in the PI expression profiles of prostate cancer and benign epithelium, PCA analysis of the 14 PIs was performed in the discovery set samples. The PCA score plots in PC1 (R^2^ = 0.562) and PC2 (R^2^ = 0.204) showed unclear clusters in cancer tissue and benign epithelium (R^2^X: 0.903[cum] and Q^2^: 0.624[cum]; Figure 4A). In contrast, OPLS-DA clearly distinguished prostate cancer from benign epithelium (R^2^X: 0.874[cum], R^2^Y: 0.735[cum] and Q^2^: 0.583[cum]; Figure 4B). The validation of a partial least squares discriminant analysis (PLS-DA) was suggestive of an excellent model (Figure S2). An OPLS S-plot relative to the proportion of PIs in cancer and benign epithelium is shown in Figure 4C. The signal intensity of m/z 863.5(PI[18:0/18:1]) and 889.5(PI[18:0/20:2]) were isolated from the S-plot as variables that strongly distinguish between prostate cancer and benign epithelium. The expression profile of the 14 PIs by the OPLS-DA was used to establish an algorithm accurately differentiating cancer from benign epithelium (Table S4). Using the algorithm with the optimal cutoff point defined as 0.5, the sensitivity and specificity for cancer diagnosis were 85.7 and 92.9%, respectively.

To evaluate whether this algorithm could be used in prostate cancer diagnosis, the expression profiles of PIs were evaluated in the validation set samples. An ROC curve calculated from this algorithm is shown in Figure 4D, with an area under the curve (AUC) of 0.964. The cutoff point of 0.5 had a sensitivity of 87.5% and a specificity of 91.7% in distinguishing prostate cancer from benign epithelium.

## Discussion

Lipids constitute diverse classes of molecules with critical functions in cellular energy storage, structure, and signaling. The risk of prostate cancer was shown to increase when particular plasma fatty acids were elevated, including myristic acid, a-linolenic acid, and eicosapentaenoic acids [22], [23]. Many individual polar lipids [24]–[27] and cholesterol-like molecules [28], [29] have been associated with the development of prostate cancer. However, it has been difficult to assess these molecules directly in prostate tissue, because most lipid analysis procedures, including conventional MS, require lipid extraction. An emerging tool, MALDI-IMS, can provide “in situ imaging”, without destroying the histological structures of biological materials, and the mapped images can be compared with the corresponding histological images [30]–[32].

Although OCT compound is commonly used to embed and preserve tissue samples, samples entirely embedded in OCT compound have been regarded as unsuitable for IMS lipid research, because contamination by OCT is easily detected by mass spectrometry and severely reduces the detection of other components [19], [20]. In this study, HR-MALDI-IMS using 9-AA as a matrix showed consistent spectra from OCT compound embedded samples in a mass range of m/z 500–1000 in negative mode. The spectral pattern and quality of these OCT-embedded samples were found equivalent to those of fresh frozen samples. This result may be important, since frozen samples are preserved in OCT compound in many clinical laboratories, allowing their use for lipid research using MALDI-IMS.

We showed that HR-MALDI-IMS had several methodological advantages compared with conventional lipidomic studies on cancer tissues. Early stage prostate cancer is often multifocal and surrounded by benign prostate epithelium and/or stroma, without the formation of a tumor mass. Moreover, since prostate cancer forms glands and the diameter of a single gland may be as small as 50 µm, conventional IMS, with a resolution greater than 50 µm, can only roughly distinguish between cancerous and other tissues. Thus, previous studies using lower-resolution IMS failed to precisely distinguish prostate cancer specific regions from benign epithelium even if they yielded potential candidates [33]–[39]. High-resolution IMS may overcome this drawback and may be useful for the analysis of heterogeneous tissues, such as prostate cancers.

We found that the composition of PIs differed markedly in prostate cancer and benign epithelium, suggesting that the differences in PI expression profiles may result from differences in the activities of several acyltransferases. These changes in PI expression profiles may not only affect cellular membrane fluidity, but also the activity of the PI3K signaling pathway [40]–[43]. Although the precise role of specific PIs in PI3K signaling remains unknown, the levels of expression of PIs containing polyunsaturated fatty acids were reported to correlate with those of PI3-phosphate [42]. Our study was preliminary and included a relatively limited number of samples. Thus, it remains to be determined whether changes in PI expression profiles and acyltransferase activities are correlated and whether they are directly related to prostate cancer development.

Several PIs, including PI(18:0/18:1), PI(18:0/20:3) and PI(18:0/20:2) were identified as possible biomarkers of prostate cancer cells. We especially focused on the distributional change of this class of molecules, not on single PIs, because the function of PIs is controlled by their comprehensive distribution. Multivariate statistical analysis, such as PCA or OPLS-DA, is regarded as a powerful tool for the holistic evaluation of complex metabolic states by clustering each sample, presuming acquired spectra as multivariable data [44]–[46]. Our biomarker algorithm for prostate cancer using OPLS-DA was able to distinguish regions of prostate cancer even in the validation set, indicating that the expression profiles of the PIs in the HR-MALDI-IMS analysis are potential biomarkers for prostate cancer diagnosis.

The expression profiles of PIs in cancer tissues were not correlated with their preoperative PSA value, Gleason score, or pathological stage (data not shown). The number of patients was small and most of the tissue samples were obtained from patients with localized and well or moderately differentiated cancers. The classifying algorithm should be verified in a larger cohort, including normal controls and patients with more aggressive disease. Moreover, the relationships between PI expression profiles and clinical outcomes remain to be determined.

Our results show that HR-MALDI-IMS can be a powerful biomarker discovery tool. The expression profile of PIs may be a candidate biomarker for prostate cancer, although this finding requires verification in a larger patient cohort and correlation with clinical outcomes. Overall, our findings suggest that the use of HR-MALDI-IMS to identify disease-specific molecular changes in prostate tissue samples will improve the critical pathology decision-making process in prostate cancer diagnosis.

## Supporting Information

Figure S1 **MS/MS spectra of common m/z species in this study.** The product ion peaks corresponding to the fatty acyl chain groups and polar head groups are described in Table S3. The x axis shows m/z. The y axis shows the signal intensity of the mass spectra. Abbreviations: LPI, lysophosphatidylinositol. PA, phosphatidic acid. PE, phosphatidylethanolamine. PI, phosphatidylinositol. Ins, inositol. sn1, fatty acid at sn-1 position. sn2, fatty acid at sn-2 position.
(PPTX)

Figure S2 **Validation based on a PLS-DA model calibrated by permutation analysis.** The model parameters for the explained variation (R^2^) and the predictive capability (Q^2^) were significant (R^2^X[cum] = 0.874; Q^2^[cum] = 0.535). The intercept values for the R^2^ and Q^2^ lines were 0.305 and −0.444, respectively.
(DOCX)

Table S1 **Signal intensity normarized to total ion current in the top 50 compounds detected in fresh frozen and OCT compound-embedded samples.**
(DOCX)

Table S2 **Common m/z species detected among the top 100 compounds in at least 12 patients in the discovery set.**
(DOCX)

Table S3 **Assignment to lipid molecular species in MS/MS negative ion mode.**
(DOCX)

Table S4 **Biomarker algorithm for prostate cancer, established using the discovery set.**
(DOCX)
