# Supplementary material for: The Expression Profile of Phosphatidylinositol in High Spatial Resolution Imaging Mass Spectrometry as a Potential Biomarker for Prostate Cancer
Source: PLoS One. 2014 Feb 28;9(2):e90242. doi: 10.1371/journal.pone.0090242 (PMC3938652; doi:10.1371/journal.pone.0090242)
Supplement: Figure S1 — MS/MS spectra of common m/z species in this study. The product ion peaks corresponding to the fatty acyl chain groups and polar head groups are described in Table S3. The x axis shows m/z. The y axis shows the signal intensity of the mass spectra. Abbreviations: LPI, lysophosphatidylinositol. PA, phosphatidic acid. PE, phosphatidylethanolamine. PI, phosphatidylinositol. Ins, inositol. sn1, fatty acid at sn-1 position. sn2, fatty acid at sn-2 position. (PPTX) [file pone.0090242.s001.pptx]

## Slide 1
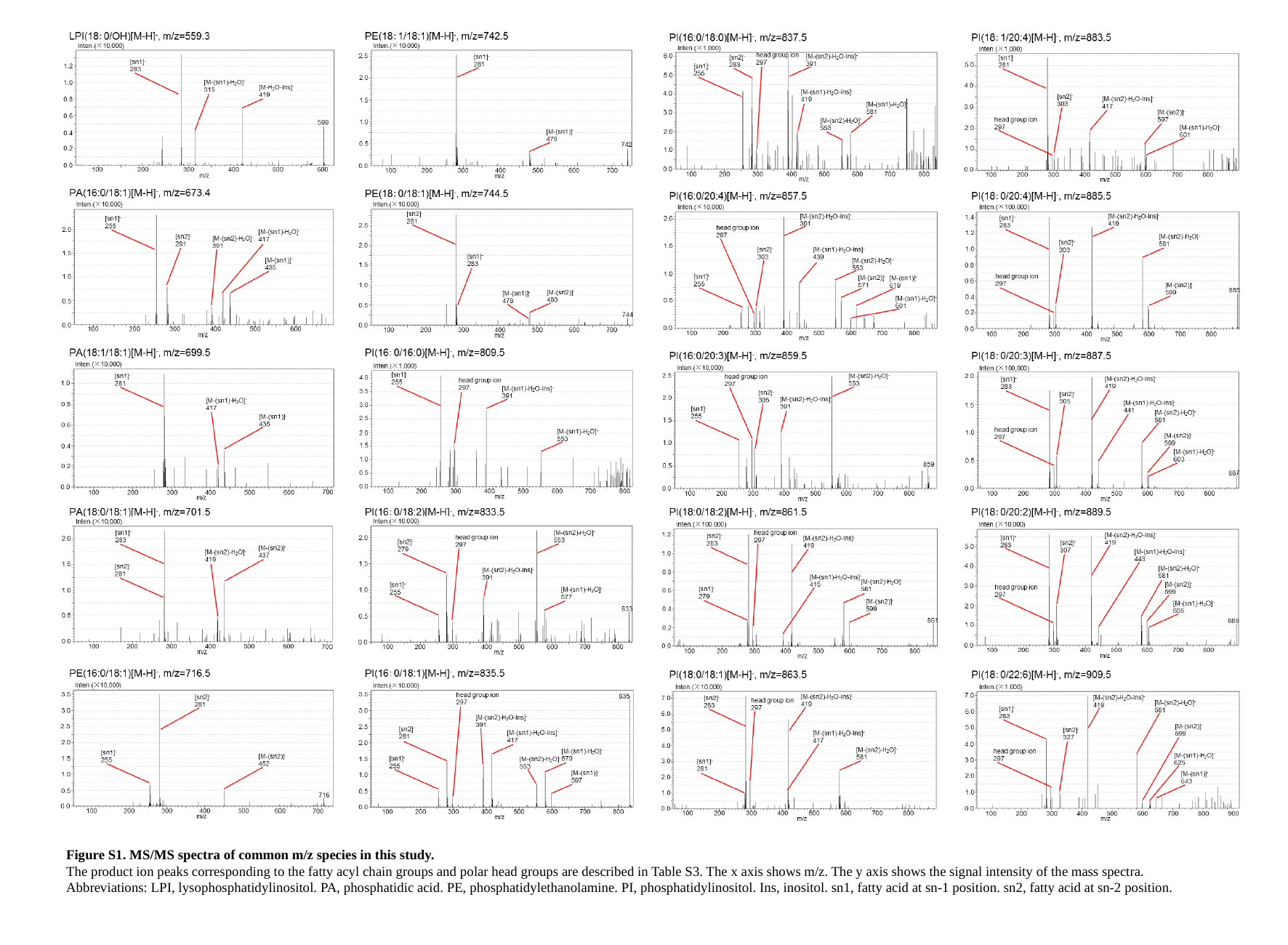

Figure S1. MS/MS spectra of common m/z species in this study.
The product ion peaks corresponding to the fatty acyl chain groups and polar head groups are described in Table S3. The x axis shows m/z. The y axis shows the signal intensity of the mass spectra. Abbreviations: LPI, lysophosphatidylinositol. PA, phosphatidic acid. PE, phosphatidylethanolamine. PI, phosphatidylinositol. Ins, inositol. sn1, fatty acid at sn-1 position. sn2, fatty acid at sn-2 position.
