# Supplementary material for: The Expression Profile of Phosphatidylinositol in High Spatial Resolution Imaging Mass Spectrometry as a Potential Biomarker for Prostate Cancer
Source: PLoS One. 2014 Feb 28;9(2):e90242. doi: 10.1371/journal.pone.0090242 (PMC3938652; doi:10.1371/journal.pone.0090242)
Supplement: Figure S2 — Validation based on a PLS-DA model calibrated by permutation analysis. The model parameters for the explained variation (R2) and the predictive capability (Q2) were significant (R2X[cum] = 0.874; Q2[cum] = 0.535). The intercept values for the R2 and Q2 lines were 0.305 and −0.444, respectively. (DOCX) [file pone.0090242.s002.docx]

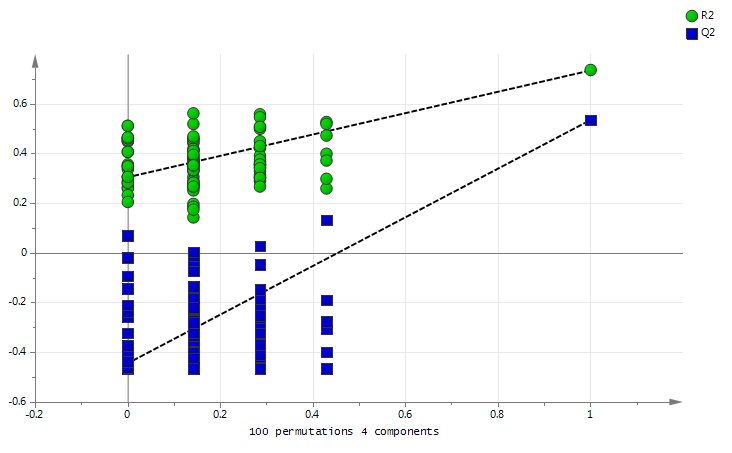


**Figure S2. Validation based on a PLS-DA model calibrated by permutation analysis.**

The model parameters for the explained variation (R^2^) and the predictive capability (Q^2^) were significant (R^2^X[cum]=0.874; Q^2^[cum]=0.535). The intercept values for the R^2^ and Q^2^ lines were 0.305 and -0.444, respectively.
