# Supplementary material for: The Expression Profile of Phosphatidylinositol in High Spatial Resolution Imaging Mass Spectrometry as a Potential Biomarker for Prostate Cancer
Source: PLoS One. 2014 Feb 28;9(2):e90242. doi: 10.1371/journal.pone.0090242 (PMC3938652; doi:10.1371/journal.pone.0090242)
Supplement: Table S1 — Signal intensity normarized to total ion current in the top 50 compounds detected in fresh frozen and OCT compound-embedded samples. (DOCX) [file pone.0090242.s003.docx]

**Table S1: Signal intensity normarized to total ion current in the top 50 compounds detected in fresh frozen and OCT compound-embedded samples.**

| **m/z** |  | **Fresh frozen samples** | |  | **OTC compound embedded samples** | |  | **p*** |
| --- | --- | --- | --- | --- | --- | --- | --- | --- |
|  |  | **Mean** | **SD** |  | **Mean** | **SD** |  | **Value** |
| 549.2 |  | 1703.4 | 2150.0 |  | 1409.8 | 1295.4 |  | 0.008 |
| 768.5 |  | 1359.9 | 1255.6 |  | 1284.5 | 1416.1 |  | 0.014 |
| 501.2 |  | 1850.9 | 2306.4 |  | 1639.5 | 1973.5 |  | 0.083 |
| 687.5 |  | 2207.3 | 2584.4 |  | 1830.9 | 2236.9 |  | 0.095 |
| 868.3 |  | 1158.7 | 1015.6 |  | 1072.8 | 267.2 |  | 0.099 |
| 575.2 |  | 2454.8 | 3437.8 |  | 1921.7 | 2458.6 |  | 0.107 |
| 702.5 |  | 1231.5 | 1393.3 |  | 1212.9 | 875.8 |  | 0.124 |
| 579.3 |  | 1841.8 | 2001.0 |  | 1691.7 | 1885.0 |  | 0.144 |
| 740.5 |  | 1373.1 | 1654.1 |  | 1327.1 | 1230.6 |  | 0.170 |
| 745.5 |  | 1309.5 | 1144.2 |  | 1369.2 | 1448.8 |  | 0.183 |
| 562.2 |  | 1699.9 | 2106.6 |  | 1535.7 | 1713.0 |  | 0.201 |
| 601.2 |  | 1471.0 | 1362.4 |  | 1585.6 | 1914.3 |  | 0.204 |
| 642.5 |  | 1493.1 | 1792.7 |  | 1576.0 | 1570.7 |  | 0.205 |
| 533.2 |  | 1348.9 | 1288.1 |  | 1269.7 | 885.8 |  | 0.211 |
| 685.4 |  | 1232.3 | 559.9 |  | 1365.2 | 1208.4 |  | 0.251 |
| 540.0 |  | 1733.0 | 1980.1 |  | 1950.6 | 2496.3 |  | 0.267 |
| 672.2 |  | 1271.5 | 1173.8 |  | 1204.5 | 343.1 |  | 0.283 |
| 742.5 |  | 2065.8 | 2681.7 |  | 1826.4 | 2245.6 |  | 0.299 |
| 599.3 |  | 2276.1 | 2785.1 |  | 1790.8 | 2160.8 |  | 0.334 |
| 542.3 |  | 1213.0 | 463.4 |  | 1225.5 | 950.6 |  | 0.343 |
| 862.5 |  | 1483.5 | 1814.9 |  | 1399.1 | 1276.0 |  | 0.361 |
| 581.2 |  | 1969.7 | 2385.9 |  | 1995.4 | 2542.1 |  | 0.361 |
| 527.2 |  | 1270.5 | 764.9 |  | 1246.2 | 902.1 |  | 0.392 |
| 576.2 |  | 2228.1 | 2746.7 |  | 2142.9 | 2510.3 |  | 0.438 |
| 674.5 |  | 1368.9 | 1403.7 |  | 1345.6 | 1034.2 |  | 0.447 |
| 556.1 |  | 1270.1 | 1296.3 |  | 1204.2 | 510.8 |  | 0.453 |
| 541.1 |  | 1297.5 | 875.2 |  | 1312.6 | 1216.8 |  | 0.460 |
| 591.1 |  | 1690.4 | 2127.7 |  | 1486.9 | 1549.5 |  | 0.476 |
| 563.0 |  | 1287.7 | 1242.5 |  | 1275.8 | 908.9 |  | 0.502 |
| 620.0 |  | 1234.1 | 1028.9 |  | 1184.8 | 434.9 |  | 0.557 |
| 564.2 |  | 1471.9 | 1523.3 |  | 1509.4 | 1723.3 |  | 0.559 |
| 606.0 |  | 3908.2 | 4689.1 |  | 3785.6 | 4522.5 |  | 0.559 |
| 612.4 |  | 1232.4 | 800.7 |  | 1284.3 | 1286.5 |  | 0.569 |
| 885.5 |  | 2757.1 | 3540.4 |  | 2873.6 | 3297.8 |  | 0.579 |
| 526.2 |  | 1268.7 | 1195.7 |  | 1305.4 | 1022.0 |  | 0.609 |
| 861.5 |  | 1621.0 | 1834.9 |  | 1775.8 | 2260.4 |  | 0.667 |
| 748.5 |  | 1274.5 | 1230.3 |  | 1230.3 | 982.5 |  | 0.685 |
| 543.1 |  | 1244.1 | 1092.0 |  | 1182.0 | 445.1 |  | 0.691 |
| 518.1 |  | 1351.0 | 1406.3 |  | 1311.2 | 1343.7 |  | 0.896 |
| 630.5 |  | 1295.9 | 999.5 |  | 1364.7 | 1263.9 |  | 0.896 |
| 701.5 |  | 1616.7 | 2051.4 |  | 1588.2 | 1777.2 |  | 0.911 |
| 577.2 |  | 3064.8 | 3501.3 |  | 3145.4 | 3651.5 |  | 0.921 |
| 525.3 |  | 1392.1 | 1553.6 |  | 1240.0 | 641.9 |  | 0.954 |
| 645.3 |  | 1234.0 | 732.2 |  | 1292.5 | 1126.2 |  | 0.964 |
| 728.5 |  | 1223.3 | 1207.9 |  | 1160.2 | 720.9 |  | 0.992 |
| 706.5 |  | 1122.4 | 431.2 |  | 1150.5 | 947.6 |  | 0.992 |

Including all detected peaks. Abbreviations: SD, standard deviation. *Mann Whitney-U test
