# Supplementary material for: The Expression Profile of Phosphatidylinositol in High Spatial Resolution Imaging Mass Spectrometry as a Potential Biomarker for Prostate Cancer
Source: PLoS One. 2014 Feb 28;9(2):e90242. doi: 10.1371/journal.pone.0090242 (PMC3938652; doi:10.1371/journal.pone.0090242)
Supplement: Table S2 — Common m/z species detected among the top 100 compounds in at least 12 patients in the discovery set. (DOCX) [file pone.0090242.s004.docx]

**Table S2: Common m/z species detected among the top 100 compounds in at least 12 patients in the discovery set.**

| **m/z** | **Number in which detected, n=14** |
| --- | --- |
| m/z591.2 | 13 |
| m/z593.2 | 14 |
| m/z599.3 | 12 |
| m/z673.4 | 12 |
| m/z687.5 | 13 |
| m/z699.5 | 14 |
| m/z701.5 | 14 |
| m/z716.5 | 14 |
| m/z742.5 | 14 |
| m/z744.5 | 14 |
| m/z747.5 | 12 |
| m/z809.5 | 13 |
| m/z833.5 | 12 |
| m/z835.5 | 14 |
| m/z837.5 | 14 |
| m/z857.5 | 13 |
| m/z859.5 | 13 |
| m/z861.5 | 14 |
| m/z863.5 | 14 |
| m/z881.5 | 13 |
| m/z883.5 | 13 |
| m/z885.5 | 14 |
| m/z887.5 | 14 |
| m/z889.5 | 14 |
| m/z909.5 | 14 |
| m/z911.5 | 13 |

Excluding matrix and isotopic peaks.
