# Supplementary material for: The Expression Profile of Phosphatidylinositol in High Spatial Resolution Imaging Mass Spectrometry as a Potential Biomarker for Prostate Cancer
Source: PLoS One. 2014 Feb 28;9(2):e90242. doi: 10.1371/journal.pone.0090242 (PMC3938652; doi:10.1371/journal.pone.0090242)
Supplement: Table S3 — Assignment to lipid molecular species in MS/MS negative ion mode. (DOCX) [file pone.0090242.s005.docx]

**Table S3: Assignment to lipid molecular species in MS/MS negative ion mode.**

| **Class** | **precursor m/z** | **MS/MS Peaks*** | **Fatty acid composition** |
| --- | --- | --- | --- |
| LPI | 599.3 | 283, 419, 315 | 18:0 |
| PA | 673.4 | 255, 281, 417, 435, 391 | 16:0/18:1 |
| PA | 699.5 | 281, 435, 417 | 18:1/18:1 |
| PA | 701.5 | 283, 437, 281, 419 | 18:0/18:1 |
| PE | 716.5 | 281, 255, 452 | 16:0/18:1 |
| PE | 742.5 | 281, 478 | 18:1/18:1 |
| PE | 744.5 | 281, 283, 480, 478 | 18:0/18:1 |
| PI | 809.5 | 255, 391, 297, 553 | 16:0/16:0 |
| PI | 833.5 | 553, 279, 391, 577, 255, 297 | 16:0/18:2 |
| PI | 835.5 | 417, 281, 391, 579, 553, 255, 597, 297 | 16:0/18:1 |
| PI | 837.5 | 391, 283, 255, 419, 581, 553, 297 | 16:0/18:0 |
| PI | 857.5 | 391, 553, 439, 571, 303, 255, 619, 297, 601 | 16:0/20:4 |
| PI | 859.5 | 553, 391, 297, 255, 305 | 16:0/20:3 |
| PI | 861.5 | 283, 419, 581, 279, 599, 297, 415 | 18:0/18:2 |
| PI | 863.5 | 283, 419, 581, 297, 417, 281 | 18:0/18:1 |
| PI | 883.5 | 281, 417, 597, 303, 297, 601 | 18:1/20:4 |
| PI | 885.5 | 283, 419, 581, 303, 599, 297 | 18:0/20:4 |
| PI | 887.5 | 419, 283, 581, 305, 441, 297, 599, 603 | 18:0/20:3 |
| PI | 889.5 | 283, 419, 307, 581, 599, 297, 443, 605 | 18:0/20:2 |
| PI | 909.5 | 419, 283, 581, 297, 327, 643, 599, 625 | 18:0/22:6 |

Abbreviations: LPI, lysophosphatidylinositol. PA, phosphatidic acid. PE, phosphatidylethanolamine. PI, phosphatidylinositol. Ins, inositol.

*arrangement - highest intensity ion (left) to lowest intensity ion (right) in the MALDI-MS/MS spectra (decimal values not included).
