# Supplementary material for: The Expression Profile of Phosphatidylinositol in High Spatial Resolution Imaging Mass Spectrometry as a Potential Biomarker for Prostate Cancer
Source: PLoS One. 2014 Feb 28;9(2):e90242. doi: 10.1371/journal.pone.0090242 (PMC3938652; doi:10.1371/journal.pone.0090242)
Supplement: Table S4 — Biomarker algorithm for prostate cancer, established using the discovery set. (DOCX) [file pone.0090242.s006.docx]

**Table S4: Biomarker algorithm for prostate cancer, established using the discovery set.**

| Formula |
| --- |
| 1.6658×10^-1^+(2.1355×10^-4^)A+(-1.4403×10^-4^)B+ (3.0291×10^-4^)C+(-1.9278×10^-4^)D  +(4.5662×10^-4^)E+(-1.8112×10^-4^)F+(-9.2727×10^-4^)G+(2.4989×10^-5^)H+(9.3707×10^-5^)I  +(-1.5560×10^-4^)J+(7.3279×10^-5^)K+(9.4254×10^-5^)L+(1.7130×10^-4^)M+(5.1632×10^-5^)N |

A-N: The signal intensity of each PI normalized to total ion current, A: LPI(18:0/OH), B: PI(16:0/16:0), C: PI(16:0/18:2), D: PI(16:0/18:1), E: PI(16:0/18:0), F: PI(16:0/20:4), G: PI(16:0/20:3), H: PI(18:0/18:2), I: PI(18:0/18:1), J: PI(18:1/20:4), K: PI(18:0/20:4), L: PI(18:0/20:3), M: PI(18:0/20:2), N: PI(18:0/22:6)
